# Cannabinol (CBN) Influences the Ion Channels and Synaptic-Related Genes in NSC-34 Cell Line: A Transcriptomic Study

**DOI:** 10.3390/cells13181573

**Published:** 2024-09-19

**Authors:** Alessandra Trainito, Claudia Muscarà, Agnese Gugliandolo, Luigi Chiricosta, Stefano Salamone, Federica Pollastro, Emanuela Mazzon, Simone D’Angiolini

**Affiliations:** 1 IRCCS Centro Neurolesi “Bonino-Pulejo”, Via Provinciale Palermo, Contrada Casazza, 98124 Messina, Italy; 2 Department of Pharmaceutical Sciences, University of Eastern Piedmont, Largo Donegani 2, 28100 Novara, Italy; federica.pollastro@uniupo.it (F.P.); 3 Department of Innovative Technologies in Medicine & Dentistry, University “G. D’Annunzio” Chieti-Pescara, Via dei Vestini, 31, 66100 Chieti, Italy

**Keywords:** cannabinol, NSC-34, transcriptomic analysis, ion channels, synaptic activities

## Abstract

Neurological disorders such as Alzheimer’s, Parkinson’s, amyotrophic lateral sclerosis, and schizophrenia are associated with altered neuronal excitability, resulting from dysfunctions in the molecular architecture and physiological regulation of ion channels and synaptic transmission. Ion channels and synapses are regarded as suitable therapeutic targets in modern pharmacology. Cannabinoids have received great attention as an original therapeutic approach for their effects on human health due to their ability to modulate the neurotransmitter release through interaction with the endocannabinoid system. In our study, we explored the effect of cannabinol (CBN) through next-generation sequencing analysis of NSC-34 cell physiology. Our findings revealed that CBN strongly influences the ontologies related to ion channels and synapse activity at all doses tested. Specifically, the genes coding for calcium and potassium voltage-gated channel subunits, and the glutamatergic and GABAergic receptors (*Cacna1b*, *Cacna1h*, *Cacng8*, *Kcnc3*, *Kcnd1*, *Kcnd2*, *Kcnj4*, *Grik5*, *Grik1*, *Slc17a7*, *Gabra5*), were up-regulated. Conversely, the genes involved into serotoninergic and cholinergic pathways (*Htr3a*, *Htr3b*, *Htr1b*, *Chrna3*, *Chrnb2*, *Chrnb4*), were down-regulated. These findings highlight the influence of CBN in the expression of genes involved into ion influx and synaptic transmission.

## 1. Introduction

The Cannabaceae family includes *Cannabis sativa* L., which is a source of fibers, oil, and different molecules useful in many sectors. The hemp has been recognized since ancient times for its medicinal properties, thanks to the presence of numerous bioactive compounds produced through secondary metabolism. These compounds include cannabinoids, terpenes, and phenolic compounds [1]. Phytocannabinoids, a specific type of terpenophenolic compound, are predominantly produced in cannabis. Five chemotypes of *Cannabis sativa* L. have been recognized on the basis of their cannabinoid profiles. In particular, chemotype III is a fiber-type plant containing high levels of non-psychoactive cannabinoids and low levels of psychoactive ones [2]. Δ^9^-tetrahydrocannabinol (Δ^9^-THC) and cannabidiol (CBD) are the main psychoactive and non-psychoactive components of the plant, respectively. In addition, tetrahydrocannabivarin (THCV), cannabinol (CBN), cannabigerol (CBG), and cannabichromene (CBC) are other major cannabinoids also produced by *Cannabis sativa* L. [3]. Currently, Δ^9^-THC and CBD are the chemical compounds most studied due to their effects on human health. Additionally, the US Food and Drug Administration (FDA) has approved their pharmaceutical preparations. Nevertheless, minor phytocannabinoids and their potential medical uses are also being investigated [4]. The pharmacological effect of phytocannabinoids is due to their interactions with the endocannabinoid system (ECS). The ECS acts as regulator of the central nervous system (CNS) by modulating synaptic and neural functions, including motor control, pain, feeding behaviors, and cognition [5]. The ECS serves as critical regulator of synaptic transmission through retrograde, non-retrograde, or autocrine mechanisms. The signaling occurs through cannabinoid receptor 1 (CB1R), cannabinoid receptor 2 (CB2R), and transient receptor potential channels (TRPs), modulating the overexcitation or inhibition of synapses [6]. Phytocannabinoids bind two receptors, CB1 and CB2, belonging to the G-protein receptors family (GPCRs) [1]. Expression studies of these two receptors have shown that the CB1 receptor is localized predominantly in the brain and in the peripheral tissues, such as the gastrointestinal tract and cardiovascular and reproductive systems. In contrast, the CB2 receptor is localized mainly in the peripheral cells, particularly those associated with the immune system [7]. Previous studies have reported that the activity of several types of ion channel is modulated by the CB1 receptor [8]. For example, it has been proven that the activation of the CB1 receptor in hippocampal astrocytes in mouse brain slices directly triggers an increase in Ca^2+^ influx in adjacent astrocytes, leading to glutamate release. This process increases the frequency of slow inward currents mediated by N-methyl-d-aspartate receptor (NMDAR) in adjacent pyramidal neurons [9]. Furthermore, in transfected AtT-20 cells, the CB1 receptor regulates the activity of G-protein-coupled inwardly rectifying K^+^ channels (GIRKs) through the GIRK activation [10].

In addition to CB1R and CB2R, phytocannabinoids can also interact with the GTP-binding-protein-coupled receptor GPR55, peroxisome-proliferator-activated receptor (PPAR), and TRP cation channels [11]. Therefore, several studies have shown that phytocannabinoids can modulate the neurotransmitter release by interacting with CB1 receptors as well as through mechanisms independent of CB receptors [12]. In contrast, most cannabinoids, are steadily studied due to their involvement with various molecular targets including ion channels and receptors [13]. Furthermore, phytocannabinoids interact with ion channels modulating Ca^2+^, Na^+^, K^+^, and Cl^−^ influx, and consequently regulate physiological processes including synaptic transmission and neuronal excitability [14]. Ion channels are essential regulators of different biological functions such as membrane permeability, apoptosis, mitosis, muscle contraction, enzymatic activity, and cellular secretion [15,16,17]. Moreover, the ion influx ensures the propagation of neuronal signals within and between neurons through synaptic transmission in excitable cells. Therefore, the pathophysiology of ion channels affects the cellular intrinsic excitability and synaptic functions, resulting in the pathophysiological signs of disease [18]. In most known neurodegenerative disorders, neuronal excitability is altered due to malfunction of ion channels, both in terms of molecular structure and function [19]. Ion channels are easily accessiblen as they are expressed on cell membranes at relatively low concentrations, making them potential targets for drug design in the specific treatment of canalopathies [18].

In our previous studies, we declared that CBG and CBD can alter the expression of genes associated with the synaptic pathway in NSC-34 cells. They influence the transcription profile of genes related to glutamate, γ-aminobutyric acid (GABA), and dopamine signaling [20]. Furthermore, we explored the transcriptomic profile of genes involved in synaptic functions in differentiated SH-SY5Y cells treated with a minor cannabinoid, Δ^8^-tetrahydrocannabinol (Δ^8^-THC). We demonstrated that treatment with Δ^8^-THC led to an up-regulation of genes associated with the glutamatergic pathway and a down-regulation of genes related to the cholinergic pathway. On the contrary, Δ^8^-THC did not influence the transcriptomic profile of GABAergic and dopaminergic pathways [21]. Finally, our group investigated the effect of cannabinerol (CBNR) in undifferentiated NSC-34 cells. CBNR was able to up-regulate genes coding for Ca^2+^, Na^+^, and K^+^ channel subunits [22]. These findings highlighted the role of phytocannabinoids in regulating the synaptic transmission to process information and respond to external and internal environmental changes [12]. In this study, we focused our analyses on CBN, which is the oxidative degradation product of Δ^9^-THC and is highly present in aged cannabis products. CBN has an agonist action on CB1 and CB2 [23]. In addition, CBN modulates several TRP channels, acting as an agonist of transient receptor potential vanilloid channels (TRPV1, TRPV2, TRPV3, and TRPV4), and is able to stimulate the TRP channels of ankyrin type-1 (TRPA1) in a powerful and effective manner [24]. CBN action on TRP channels activates Ca^2+^-dependent processes in cells, stimulating the Ca^2+^ influx [24,25].

The motoneuron-like cell line NSC-34 is a hybrid cell line produced by the fusion of neuroblastoma with mouse motoneuron-enriched primary spinal cord cells. In this cell line, there are two cell populations: smaller undifferentiated cells capable of division and bigger multinucleate cells. In addition, this cell line expresses numerous features of motor neurons, such as neurofilament triplet proteins, acetylcholine synthesis, storage and release, and the generation of action potential [26]. Thus, undifferentiated NSC-34 cells are also used as in vitro models for studying the pathophysiology of motor neurons, such as in multiple sclerosis, amyotrophic lateral sclerosis, and spinal cord injury [27,28]. Moreover, NSC-34 cells responded to agents that affect cytoskeletal organization, axonal transport, and voltage-gated ion channels. In this context, NSC-34 is a good model to investigate compounds acting through ion channels [29].

Thus, we treated undifferentiated NSC-34 cells with different concentrations of CBN (5, 10, 20, 50, and 100 µM) in order to investigate how this phytocompound can alter the transcriptomic profile of cell line genes involved to synaptic and ion channel pathways.

## 2. Materials and Methods

### 2.1. Synthesis of CBN

Non-psychoactive *Cannabis sativa* L. was purchased from Canvasalus Srl (Monselice, Italy), and a certified sample (Cs-CBD/03/2021) is stored in Novara laboratories. CBN was obtained from 100 mg of CBD, which was extracted from *Cannabis sativa* L., belonging to the chemotype III in accordance with the protocol described by Pollastro et al. [30]. CBD was extracted and purified from non-woody *Cannabis sativa* L. aerial parts using HPLC. The pure oil of CBN was identified using 1H 400 MHz NMR spectra with Bruker 400 spectrometers (Bruker^®^, Billerica, MA, USA). Subsequently, chemical shifts related to the remaining solvent signal (CDCl3: δH = 7.26) were measured. To perform low-pressure chromatography, silica gel 60 (70–230 mesh) was acquired from Macherey-Nagel (Düren, Germany). Purifications were tracked by staining with 5% H_2_SO_4_ in EtOH and heating employing TLC. Aldrich (Darmstadt, Germany) provided the chemical reagents and solvents used which were applied without further purification unless expressly specified. HPLC JASCO Hichrom, 250 mm × 25 mm, silica UV–vis detector-2075 plus (Jasco, Tokyo, Japan) was used.

### 2.2. NSC-34 Cells Culture and Treatment

The NSC-34 motoneuron-like cell line was provided by Cellutions Biosystem Inc., Cedarlane (Burlington, ON, Canada) and was cultured in Dulbecco’s Modified Eagle’s Medium (DMEM) high glucose spiked with 1% L-glutamine, 1% penicillin/streptomycin and 10% Fetal Bovine Serum (FBS). The cells were maintained at 37 °C in 5% CO_2_/95% air humidified atmosphere and were subcultured every 2–3 days.

NSC-34 cells were treated for 24h with different concentration of CBN (5, 10, 20, 50, and 100 µM). CBN was dissolved in dimethyl sulfoxide (DMSO), diluted in Phosphate-Buffered Saline 1 × (PBS), and added to the final concentrations in the medium (the final concentration of DMSO was <0.1%) [31]. The untreated NSC-34 cells (CTRL) were incubated with the same concentration of DMSO (DMSO < 0.1%). All reagents were provided by Sigma-Aldrich, St. Louis, MO, USA.

### 2.3. MTT Assay

In order to evaluate cell viability, NSC-34 cells were plated in 96-well microplates at a density of 20 × 10^3^ cells per well, then treated with CBN at different concentrations (5, 10, 20, 50, and 100 µM) for 24 h, and an MTT assay was assessed.

After treatment, cells were incubated with fresh medium supplemented with 0.5 mg/mL MTT reagent (Thiazolyl Blue Tetrazolium Bromide) (Sigma-Aldrich, St. Louis, MO, USA) at 37 °C for 4 h and then with the addition of HCl/isopropanol solution 0.04 N for 1 h. After incubation, insoluble formazan crystals were dissolved, and the absorbance was measured at 570 nm using a spectrophotometer (BioTek Synergy H1 microplate reader, BioSPX, Beersel, Belgium). Each experimental condition was replicated 8 times.

### 2.4. Library Preparation

NSC-34 cells were seeded in 6-well plates at a density of 5 × 10^5^ cells in 2 mL of medium per well and treated with different doses of CBN (5, 10, 20, 50, and 100 µM). After collecting the cell pellet, the total RNA was extracted using a Maxwell^®^ RSC simplyRNA Cells Kit (Promega, Madison, WI, USA) as suggested by the protocol. Library cDNA preparation was carried out with the TruSeq^®^ RNA Exome protocol, following the instructions, as previously reported by Artimagnella et al. [22]. A TapeStation 4150 instrument and D1000 screentape were used to validate the quality of the library (Agilent, Richardson, TX, USA). A NextSeq 500/550 Mid Output Reagent Kit v2 (300 cycles) was used for sequencing with the Illumina instrument NextSeq 550Dx. The reagents used for library preparation were provided by Illumina, San Diego, CA, USA. The run was performed in paired-end mode.

### 2.5. Bioinformatic Analysis

To assess the quality of the reads analyzed in this study, we employed FastQC v.0.12.0 (Babraham Institute, Cambridge, UK) (FastQC. A Quality Control Tool for High Throughput Sequence Data. Available online: https://qubeshub.org/resources/fastqc accessed on 7 June 2024). The reads obtained were subsequently processed using Trimmomatic v.0.40-rc1 (Usadel Lab, Aachen, Germany) [32], allowing us to remove adapters and readings exhibiting low-quality scores. Following this preprocessing step, the residual reads were aligned to the hg38 v39 reference genome from GENCODE utilizing the STAR RNA-seq aligner 2.7.10a_alpha_220207 (New York, NY, USA) [33]. Post-alignment, transcript counts per gene were derived using HTSeq v.0.13.5 [34]. Differential gene expression analysis was conducted using the DESeq2 library v.1.36.0 [35] within R v.4.2.0 (R Core Team). To mitigate the false-positive rate, the *p*-values from the DESeq2 analysis were corrected using the Benjamini–Hochberg method with a *q*-value threshold of 0.05. Subsequently, the list of differentially expressed genes (DEGs) was subjected to gene ontology (GO) overrepresentation analysis (ORA) via the R package clusterProfiler v.4.4.3 [36]. The *p*-value obtained from ORA analysis was corrected to reduce the false positive number, and the ontologies with a *q*-value ≤ 0.05 were considered for the subsequent analysis. Furthermore, detailed examination of specific biological processes was facilitated through the AmiGo2 database [37]. Complementary insights into the pathways perturbed by DEGs were acquired utilizing the KEGG database [38].

### 2.6. Western Blot Analysis

Protein expression of CB1R, and NMDA receptor 2B in NSC-34 cells treated with different concentrations of CBN (5, 10, 20, 50, 100 µM), were evaluated by Western blot analysis. The RIPA buffer supplemented with Protease Inhibitor Cocktail (Thermo Scientific™, Waltham, MA, USA) was used for total protein extraction, and their concentrations were analyzed using the Bradford assay (Bio-Rad, Hercules, CA, USA). A quantity of 25 µg of proteins from each sample were denatured at 95 °C for 5 min, and were separated by SDS-polyacrylamide gel electrophoresis (SDS-PAGE) and transferred to PVDF membranes (Immobilon–P, Millipore, Burlington, MA, USA). Then, the blots were blocked with 5% skim milk in Tris-buffered saline 1X containing 0.1% Tween 20 (TBS-T 1X) for 1h at room temperature. The blots were incubated at 4 °C overnight with anti-CB1 Receptor (1:1000; Cell Signaling Technology, Danvers, MA, USA), and anti-NMDAR2B (1:500; Alomon Labs, Jerusalem, Israel). After washing with TBS-T 1X, the membranes were incubated with anti-rabbit (1:2000; Santa Cruz Biotechnology Inc., Dallas, TX, USA). The relative expression of protein bands was visualized using an enhanced chemiluminescence system (Luminata Western HRP Substrates, Millipore, Burlington, MA, USA), and protein bands were acquired with the ChemiDoc™ XRS+ System (Bio-Rad, Hercules, CA, USA). Bands were quantified using ImageJ v.1.53t software. The molecular weight of the unknown band obtained was estimated by plotting the logarithm of protein molecular weight vs. the relative mobility (Retention Factor R_f_) of the protein [39]. Thus, the bands were selected based on the molecular weight provided by supplier. The originals membranes are available in Supplementary Materials.

### 2.7. Statistical Analysis

Statistical analysis was performed using Graph-Pad Prism software v.9.5.1 (Boston, MA, USA). The results were reported as mean ± Standard Deviation (SD) and were analyzed by one-way ANOVA test comparing the mean between each experimental group, and the Bonferroni post hoc test for multiple comparisons was performed. We considered statistically significant each adjusted *p*-value ≤ 0.05.

## 3. Results

### 3.1. Evaluation of Cell Viability after CBN Treatment

In order to assess the cytotoxic effect of CBN, NSC-34 cells were exposed for 24 h with different doses of compound in the range of 5 µM to 100 µM, and an MTT assay was carried out.

**Figure 1:**
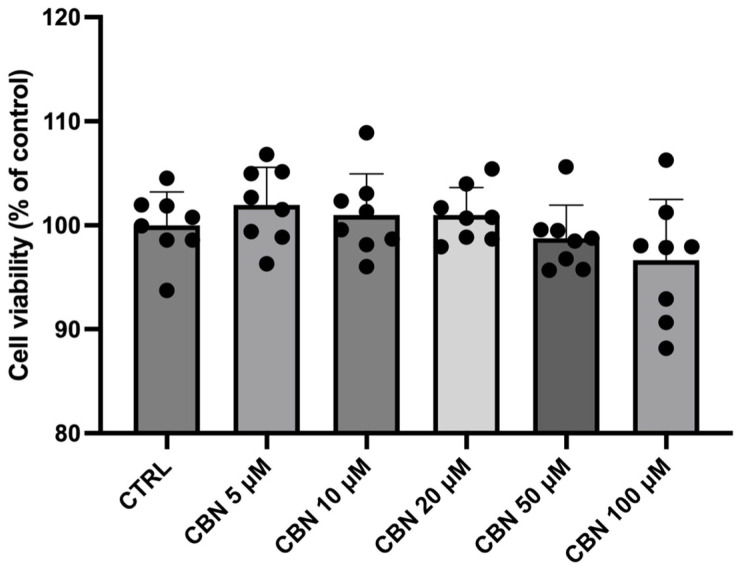
Effect on NSC-34 cell viability after CBN treatment for 24 h at different concentrations (5, 10, 20, 50, and 100 µM) compared to untreated NSC-34 cells (CTRL). Results are expressed as relative percentages to CTRL. Data are means ± SD from eight independent experiments. The black dots represent the individual values obtained from each repetition of spectrophotometric analysis.

The treatment of NSC-34 cells to different CBN concentrations did not cause any significant variation in the spectrophotometric absorbance resulting from the viability assay. According to our results, CBN did not decrease the cell viability of treated cells in comparison to untreated cells (CTRL). As reported in Figure 1, the compound resulted as not cytotoxic, even at the highest concentration.

### 3.2. Transcriptomic Analysis

**Figure 2:**
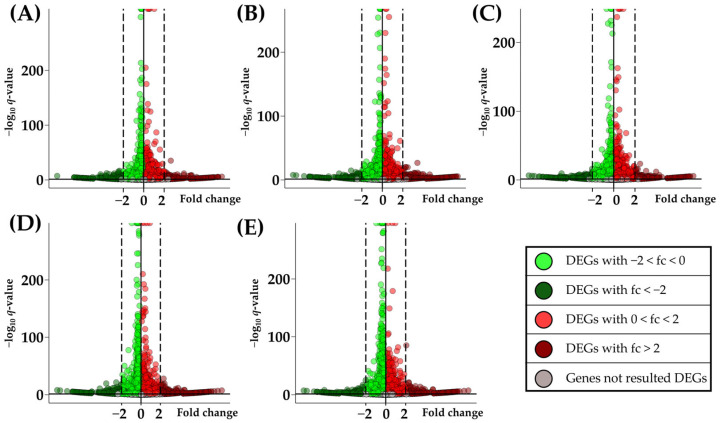
In the volcano plots are reported all the genes inspected in each comparison. Each volcano is marked with different letters that indicate the different comparisons performed: (**A**) for CTRL vs. CBN 5 µM, (**B**) for CTRL vs. CBN 10 µM, (**C**) for CTRL vs. CBN 20 µM, (**D**) for CTRL vs. CBN 50 µM, and (**E**) for CTRL vs. CBN 100 µM. In the *x* axis is reported the fold change while in the *y* axis is reported the −log10 *q*-value with a horizontal line that marks the statistical threshold 0.05.

The effect of CBN in NSC-34 cells was evaluated through the differentially expressed gene (DEG) inspection obtained by the different comparisons performed. We compared the control group (CTRL) against the groups treated with 5 different doses of CBN: 5 µM (CBN 5 µM), 10 µM (CBN 10 µM), 20 µM (CBN 20 µM), 50 µM (CBN 50 µM), and 100 µM (CBN 100 µM). Our comparisons were used to discover how different doses of CBN can alter the physiologically cellular processes. For each comparison, the Wald test was performed for each gene, and the resulting *p*-values were adjusted using the Benjamini–Hochberg post hoc correction to obtain the *q*-value. We considered as DEGs all the genes with a *q*-value ≤ 0.05. All the genes analyzed for every comparison with the associated fold change, *p*-value, and *q*-value are available in Table S1. In the volcano plots represented in Figure 2 is reported an overview of the genes resulting in up-regulated, down-regulated, or non-DEGs. The term ‘up-regulated’ denotes the DEGs exhibiting a statistically validated elevation in expression within the sample treated with CBN, while ‘down-regulated’ DEGs indicate a statistically validated reduction in expression within the sample treated with CBN compared to the CTRL group.

Starting from the DEGs resulting from the analysis reported above, we performed the gene ontology ORA to better investigate how each dose of CBN can alter the physiologically cell conditions. Inspection of the biological processes (BPs), molecular functions (MFs), and cellular components (CCs) allows us to obtain an overview of the global alterations resulting from our DEGs. All the DEGs with a fold change ≤−2 or ≥2 have been isolated for ORA to highlight just the ontologies that include a list of DEGs with a strong alteration caused by treatment with CBN.

In Table 1 are reported, for each comparison, the number of DEGs with a fold change ≤−2 or ≥2 followed by information about the number of ontologies over-represented for that comparison.

**Figure 3:**
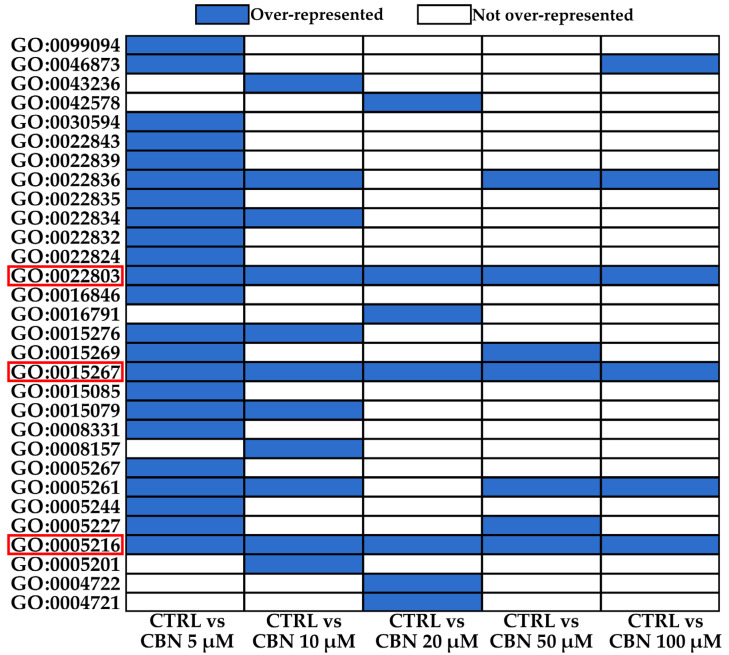
The graph shows the different MFs over-represented in the *y* axis and the comparison in the *x* axis. Each coloured cell highlights a specific MF over-represented in the comparisons. The MFs highlighted by red rectangles were over-represented in all the comparisons.

All the results obtained from ORA with ontologies over-represented, *q*-value associated, and list of DEGs that fall into each ontology are reported in Table S2. According to the information shown in Table 1, we can see that the number of DEGs with an extreme fold change is similar in each comparison (~900). ORA highlighted that not all the comparisons contain over-represented BPs (CTRL vs. CBN 5 µM and CTRL vs. CBN 20 µM do not contain any over-represented BPs), and the same goes for CCs (CTRL vs. CBN 20 µM does not contain any over-represented CCs). To investigate the over-represented ontologies shared by different comparisons, we cross-referenced all the MFs reported in Table 1, and the result of this analysis is reported in the graph shown in Figure 3.

**Figure 4:**
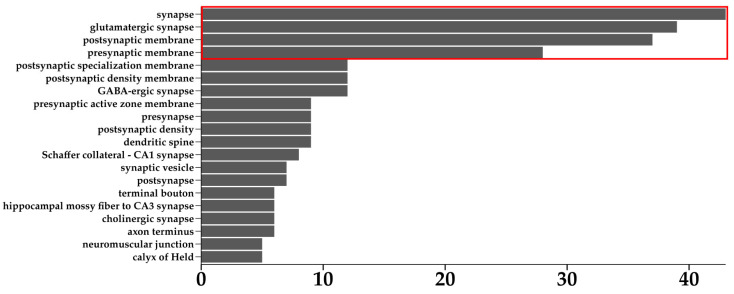
The bar plot shows the number of DEGs involved in the different terms and sub terms. The red box highlights the terms and sub terms that include the higher part of the DEGs related to the MF “ion channel activity”.

Figure 3 shows that across all the over-represented MFs, just three of them are over-represented in all the comparisons. The three above-mentioned MFs are GO:0005216 “ion channel activity”, GO:0015267 “channel activity”, and GO:0022803 “passive transmembrane transporter activity”. Each of the abovementioned MFs is related to channel activity, and this shows that the treatment with each dose of CBN can strongly alter the processes related to each MF. We can talk about “strong alteration” depending on whether this result was obtained starting from the DEGs with an associated extreme fold change. Among the three MFs reported above, we investigated if these were also over-represented in the ORA performed on all the DEGs without any filter of the fold change. This additional analysis was crucial to take a broader view of these MFs and obtain information about all the DEGs involved in them, as well as those with a non-extreme fold change. All the results of this second ORA, performed using all the DEGs without any filter based on the fold change, all the ontologies, and related *p*-value and *q*-value are reported in Table S3. Of the three MFs mentioned, just one was also over-represented in every comparison for the ORA performed, starting from all the DEGs without any filter based on the fold change; this MF was “ion channel activity”. Considering this over-representation always present in our analysis, we decided to focus our attention on the DEGs involved in it. Across all the comparisons, the DEGs involved in the MF “ion channel activity” are 195 and are reported in Table S4 with the associated fold change for each comparison. Since the propagation of the neuronal signal across the axons is ensured by the control of the ion flows, the ion channels represent key elements in the control of the synaptic transmission, so we inspected among the 195 DEGs mentioned above and reported those involved in synaptic activity. From database MGI [40], we imported all the mouse genes related to synaptic activity and cross-referred them with the 195 related to the MF “ion channel activity” highlighted by our analysis. Among the 195 DEGs inspected, 83 were involved in synaptic activities at different levels. All of the results related to the analysis just mentioned are available in Table S5, where it is reported all the DEGs involved in synaptic activity with associated synaptic terms and subterms. The 83 DEGs mentioned fall into a total of 40 terms and subterms related to synaptic activity; all the terms with the associated number of DEGs are reported in the bar plot shown in Figure 4.

Different DEGs are associated not only with the main term “synapse” but, in addiction, with other different subterms. As observable in Figure 4, the terms and subterms that include the vast majority of DEGs involved in synaptic activity are “synapse”, “glutamatergic synapse”, “postsynaptic membrane”, and “presynaptic membrane”. We have filtered out all the DEGs involved in the above-mentioned terms, obtaining a list of 69 DEGs with the fold change for each comparison performed; all this information is reported in Table S6. Among the DEGs reported in Table S6, we filtered out different groups of genes that could be the most interesting for our study. The first class of genes considered from the list of DEGs reported in Table S6 was composed of all the genes resulting in DEGs in every comparison with the same trend of expression. Up-regulation or down-regulation of these genes suggests that the treatment of CBN, at every dose, has an impact on the expression of these genes. All the above-discussed DEGs are reported in Table 2.

In addition to the DEGs reported in Table 2, there are two genes that were DEGs in all comparisons but not with the same trends for the different comparisons; these DEGs were *Cacna1a* and *Itpr1*, consultable in Table S6. Genes reported in Table 2 encode proteins that play a role in the regulation of ion flux, neurotransmitter release, and synaptic transmission. In particular, *Cacna1b* and *Cacna1h* were up-regulated, and they encode calcium voltage-gated channel subunit alpha 1B and subunit alpha 1H, respectively. In contrast, the calcium voltage-gated channel subunit beta 4 (*Cacnb4*) and calcium voltage-gated channel subunit gamma 2 (*Cacng2*) were down-regulated in all comparisons. The *Gjc1* gene, encoding for Gap junction gamma 1 protein, was down-regulated. The subunit 5 of the ionotropic glutamate receptor of the kainite type (*Grik5*) was up-regulated. In contrast, subunit 1 of N-Methyl-d-aspartate NMDA receptors (*Grin1*) was down-regulated. The 3a and 3b subunits of 5-hydroxytryptamin receptor (*Htr3a* and *Htr3b*) were highly down-regulated, and genes coding for potassium voltage-gated channel (*Kcnc3* and *Kcnd1*) were up-regulated. Finally, the gene coding for synaptosomal-associated protein 25 kDa (*Snap25*) was down-regulated. All these genes found differentially expressed in our study showed the same regulation trend at all doses.

To investigate the difference among the effects caused by the treatment at different doses with CBN, we filtered out all the DEGs differentially expressed in four out of five comparisons performed. A list of DEGs is reported in Table 3.

The genes that resulted DEGs in four out of five comparisons showed the same trend for all doses. There are not any genes reported as DEGs in the four out of five comparisons that do not take the same trend of expression, as observable in Table S6. The genes encoding for subunits of the neuronal acetylcholine receptor, *Chrna3* and *Chrnb2*, were down-regulated, except for CTRL vs. CBN 10 µM and CTRL vs. 100 µM, respectively, and *Chrna4* was up-regulated save for CTRL vs. CBN 20 µM. The cannabinoid receptor 1 gene (*Cnr1*) was up-regulated in all comparisons except for CTRL vs. CBN 50 µM. The gamma-aminobutyric acid type A receptor subunit alpha5 gene (*Gabra5*) was highly up-regulated save for CTRL vs. CBN 10 µM. The subunits 1 and 4 of the ionotropic glutamate receptor of the kainite type (*Grik1* and *Grik4*) were up-regulated and down-regulated, respectively. The subunit beta 1 of 5-hydroxytryptamin receptor (*Htr1b*) was highly down-regulated except for CTRL vs. CBN 20 µM. The potassium voltage-gated channel gene *(Kcnk1*) was down-regulated except for CTRL vs. CBN 50 µM. Finally, sodium channel protein type 8 subunit alpha (*Snc8a*) was up-regulated except for CTRL vs. CBN 20 µM. Moreover, as shown in Table 3, the genes resulting in DEGs in four out five comparisons were always DEGs in CTRL vs. CBN 5 µM.

Considering the different comparisons performed and the importance of discovering which dose can have the most interesting impact on synaptic activity, we filtered out another class of DEGs. The last class of DEGs considered for additional analysis was composed by all the genes that showed up as deregulated in at least two comparisons and that, from a certain dose or up to a certain dose, were up- or down-regulated. These above-mentioned DEGs are reported in Table 4. In particular, *Cacng8*, *Slc17a7*, and *Tspoap1* were up-regulated starting from the dose of 20 µM up to 100 µM. *Kcnd2* was up-regulated at the doses of 50 µM and 100 µM. *Cdk5* and *Kcnj4* were up-regulated at the doses 5 µM and 10 µM. Lastly, *Chrnb4* was down-regulated from the dose of 5 µM to 20 µM.

In the list of DEGs reported in Table 4, “*Chrnb2*” and “*Grik1*” should have also been shown. These DEGs are not reported because their expression is already shown in Table 3, but considering their fold change in the different comparisons, they meet the requirements to be shown in both Table 3 and Table 4.

### 3.3. Proteins Expression Levels Analysis

**Figure 5:**
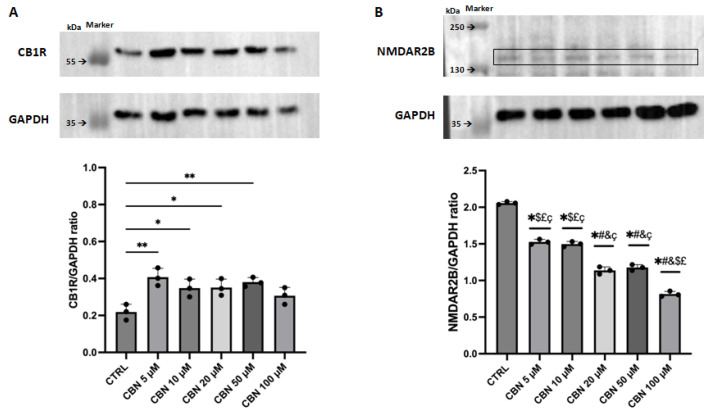
Western blot analysis of proteins related to ion channel and synaptic activity. We reported the concentration of protein levels at all concentrations and the bands from WB membranes. The statistical analysis was performed by comparing the mean between each experimental group. (**A**): CB1R concentration compared to the housekeeping protein GAPDH. * *p* < 0.05, and ** *p* < 0.01 indicate significant difference vs. CTRL. (**B**): NMDAR2B expression levels compared to the housekeeping protein GAPDH. * *p* < 0.0001 indicates significant difference vs. CTRL. ^#^ *p* < 0.0001 indicates significant difference vs. CBN 5 µM. ^&^ *p* < 0.0001 indicates significant difference vs. CBN 10 µM. ^$^ *p* < 0.0001 indicates significant difference vs. CBN 20 µM. ^£^ *p* < 0.0001 indicates significant difference vs. CBN 50 µM. ^ç^ *p* < 0.0001 indicates significant difference vs. CBN 100 µM. In the blot, the black square indicates the band related to NMDAR2B.

In order to assess the potential role of CBN in regulating the expression of genes associated with ion channel and synaptic activity, we evaluated the protein levels of CB1R and NMDAR2B, as reported in Figure 5.

CB1R was statistically increased from the dose of 5 to 50 µM vs. CTRL, with a greater significance to the concentration of 5 µM. In contrast, the statistical analysis across the different concentrations did not reveal any significant difference. Conversely, the subunit NMDAR2B of the NMDA receptor was statistically decreased at all tested doses of CBN. The statistical analysis comparing data across different doses showed that CBN could have a dose-dependent effect on NMDAR2B expression. The original membranes were available in Supplementary Figures S1 and S2.

## 4. Discussion

Ion channels and synaptic transmission act a key role in many physiological processes and disease mechanisms. Numerous prevalent neurodegenerative disorders are characterized by alterations in neuronal excitability, which are attributable to dysfunctions in the molecular and functional mechanisms of ion channels [19]. Neurodegenerative diseases, such as Alzheimer disease (AD), Parkinson disease (PD), Huntington disease (HD), amyotrophic lateral sclerosis (ALS), and frontotemporal dementia (FTD), are associated with alterations of neuronal transmission owing to degeneration or dysfunction of ion channels and synapses. In this context, several studies aim to improve synaptic function and delay neurodegenerative processes using ion channel- and synapse-targeting therapies [41]. Phytocannabinoids are widely studied as potential therapeutic compounds for disorders originating throughout the body, especially in excitable tissues like nerves and muscles [42]. The therapeutic effects of the major phytocannabinoids are due to their interaction with the ECS and ion channels, which regulate synaptic transmission and neuronal excitability [11].

We focused our study on CBN and its effects on an undifferentiated cell line of motoneurons. The aim was to observe the alterations in gene expression after 24 h of incubation with different concentrations of the CBN phytocompound (5, 10, 20, 50, and 100 µM).

After treatment of the NSC-34 cell line with CBN, the MTT assay was carried out, and the spectrophotometric absorbance showed that the phytocompound was not cytotoxic at tested concentrations (5, 10, 20, 50, and 100 µM), as reported in Figure 1. Our study focused on transcriptomic analysis that allowed us to explore all the gene expression alterations caused by the expositions at the different doses of CBN. We compared the control group (CTRL) against the groups treated with five different doses of CBN: 5 µM (CBN 5 µM), 10 µM (CBN 10 µM), 20 µM (CBN 20 µM), 50 µM (CBN 50 µM), and 100 µM (CBN 100 µM). The analysis of DEGs for each comparison was conducted using gene ontology ORA. DEGs with a fold change of ≤−2 or ≥2 were selected to better understand how each dose of CBN could strongly alter the physiological condition of the cells. The number of DEGs with extreme fold change for each comparison is reported in Table 1. ORA analysis performed on the above-mentioned DEGs showed that all comparisons contain at least one over-represented MF, and the cross-reference revealed that three MFs were shared with all doses, as shown in Figure 3. Surprisingly, the shared MFs were “ion channel activity”, “channel activity”, and “passive transmembrane transporter activity”. Our results suggest that the CBN is able to regulate the genes involved in these MFs. In order to have a broader view of these MFs and obtain information about all the DEGs involved in them, we investigated if these were also over-represented in the ORA performed on all the DEGs without any filter based on the fold change, and we highlighted the over-representation in every comparison of the MF “ion channel activity”. The genes resulting in DEGs involved in the MF “ion channel activity” were 195 DEGs across all comparisons.

Using database MGI [43], we found that 83 out of the above-mentioned 195 DEGs were associated with synapse terms and sub terms, in particular, “synapse”, “glutamatergic synapse”, “postsynaptic membrane”, and “presynaptic membrane”, as reported in Figure 4. Analyzing all the genes resulting in DEGs in every comparison with the same trend of expression reported in Table 2, we found four genes coding for calcium voltage-gated channel subunits; in particular, *Cacna1b* and *Cacna1h* were up-regulated, and *Cacnb4* and *Cacng2* were down-regulated. The calcium voltage-gated channel gene family (*Cacna*) encodes the alpha subunits of voltage-gated calcium channel complexes (VGCCs). VGCCs are crucial regulators of calcium influx into cells and have important roles in neuronal excitability, synaptic transmission, and plasticity [44]. The dysregulation of these complexes is associated with the pathogenesis of conditions such as migraine, epilepsy, cerebellar ataxia, dystonia, and cerebellar atrophy [45]. The influx of calcium ions is crucial for the regulation of cell signaling, which results in neurotransmission, muscle contraction, and gene expression regulation [46]. *Cacna1b* encodes the alpha-1B subunit of the N-type voltage-dependent calcium channel (Ca_v_2.2), and regulates calcium influx in neurons at the presynaptic terminals, modulating neurotransmitter release and consequentially synaptic transmission [47]. *Cacna1h* encodes the alpha-1H subunit of the T-type voltage-dependent calcium channel (Ca_v_3.2), and, forming the pore of the channel, plays a role in regulating muscle contraction, synaptic transmission, secretion, and gene expression [48,49]. The *Cacnb4* gene encodes the beta4 subunit, a non-pore-forming modulatory subunit of calcium channels. Beta4 is an auxiliary subunit of P/Q-type calcium channels, and regulates the kinetics, amplitude, and voltage-dependence of calcium influx by modulating the gating properties of high-voltage-gated calcium channels [50]. *Cacng2* is an auxiliary subunit of AMPA (α-amino-3-hydroxy-5-methyl-4-isoxazolepropionic acid) glutamate receptors; this transmembrane regulatory protein modulates excitatory neurotransmission, receptor function, and distribution at the synapse [51].

*Gjc1* coding for connexin 45 (CX45) was down-regulated by CBN treatment in all doses tested. This gene is a member of the connexin family and whose encoded protein is a component of hemichannels, which constitute the gap junctions. The role of CX45 is not fully studied in CNS and related disorders. Conversely, it was seen that mutations in *Gjc1* could predispose people to congenital heart disease (CHD) and arrhythmias [52]. Moreover, *Snap25*, coding for synaptosomal-associated protein of 25 kDa, was down-regulated in all comparisons. The role of the SNAP25 protein is associated with VAMP (vesicle-associated membrane protein) and syntaxin proteins, which together constitute the SNARE complex. The above-mentioned complex is essential for the exocytosis of synaptic vesicles and, thereby, for synaptic transmission [53].

Exposure to CBN in all the tested doses led to an up-regulation of *Grik5* encoding for the subunit 5 of the ionotropic glutamate receptor belonging to the kainate type, and down-regulation of *Grin1* encoding for the subunit 1 of N-Methyl-D-aspartate (NMDA) ionotropic glutamate receptors. There exist three main types of ionotropic glutamate receptors: kainate receptors, N-methyl-D-aspartate (NMDA), and alpha-amino-3-hydroxy-5-methyl-4-isoxazolepropionic acid (AMPA) [54]. *Grik5* is a member of the glutamate kainate receptor family and plays an important role in neural development and neuropsychiatric disorders [55]. *Grin1* encodes a subunit of the ionotropic glutamate NMDA receptor (NMDAR1B) and regulates many physiological and pathophysiological processes [56]. The NMDA receptor is a heterotetrametric channel consisting of two subunits of NR1 and two subunits of NR2. The subunit NR2 (NMDAR2B) is encoded by the *Grin2* gene [57]. The analysis of total western blot reported in Supplementary Figure S2, showed non-specific bands for the polyclonal antibody NMDAR2B, so we estimated the unknown molecular weight of our band by plotting the logarithm of protein molecular weight vs. the relative mobility [39]. Using this method, we supposed that the band of 150 kDa was our target protein, NMDAR2B. As reported in Figure 5B, the NMDAR2B subunit decreased in all concentrations of CBN tested in a dose-dependent manner. However, remember that protein expression depends on several factors; therefore, the transcriptional profile is not always directly related to protein expression [58]. Glutamate is a critical neurotransmitter in the CNS and is essential for various cognitive functions such as memory, neuronal communication, development, plasticity, and learning [59]. Glutamate binds ionotropic and metabotropic receptors to carry out its action in the CNS. The activation of ionotropic glutamate receptors results in a flow of Na^+^, K^+^, and Ca^2+^ ions across the neuronal membrane, leading to the propagation of neural signals [60].

Regarding serotonin receptors, *Htr3a* and *Htr3b* were highly down-regulated in all doses tested of CBN. *Htr3a* and *Htr3b* encode for 3a and 3b subunits of 5-hydroxytryptamin receptor (5-HT_3_R). The 5-HT_3_R is a cation-selective ligand-gated ion channel mediating neuronal depolarization and excitation in the central and peripheral nervous systems [61]. 5-hydroxytryptamin is an important neurotransmitter with various functions, including regulation of appetite, sleep, mood, cognitive functions, and pain. Furthermore, *Htr3a* and *Htr3b* gene-related polymorphisms are associated with genetic predisposition to musculoskeletal pain, depression disorders, and increased psychological conditions of dementia in patients with AD [62].

Concerning potassium flux, *Kcnc3* and *Kcnd1* results highly up-regulated by CBN treatment at all doses (5, 10, 20, 50, and 100 µM). *Kcnc3* encoding for a subunit of the voltage-gated potassium channel (Kv3.3) and shows N-type inactivation. Voltage-gated potassium channels (Kv3) play an important role in the rapid repolarization of quick-firing brain neurons, and they are diffusely expressed throughout the brain, including the hippocampus, cortex, cerebellum, and auditory brainstem [63]. Kv3 channels are only activated when the action potential has reached its peak and are deactivated rapidly after the rise. This mechanism of action has suggested that Kv3 channels contribute to the repolarization of the action potential and play a role in determining its duration [64]. In contrast, *Kcnd1* encodes a subunit of the voltage-dependent potassium channel (Kv4). The four members of the Kv4 channel family are responsible for native, rapidly inactivating (A-type) currents described in neurons [65].

The genes mentioned above were differentially expressed in all comparisons, and all genes had the same expression trend, either all up- or down-regulated by different doses of CBN (5, 10, 20, 50, and 100 µM). The expression levels of these genes associated with synaptic activity and ion channels did not show to have an increasing or decreasing trend; therefore, CBN does not appear to have a dose-dependent effect on the expression of these genes.

From the analysis of DEGs associated with synaptic activity, the terms showing the same regulatory trend and expressed in four out of five concentrations of CBN were filtered as reported in Table 3. As shown in Table 3, *Chrna4*, *Cnr1*, *Gabra5*, *Grik1*, and *Snc8a* were up-regulated in four out of five concentrations. In contrast, *Chrna3*, *Chrnb2*, *Grik4*, *Htr1b*, and *Kcnk1* were down-regulated in four out of five concentrations. In particular, *Chrna4*, *Chrna3*, and *Chrnb2* encode the alpha and beta subunits of the cholinergic acetylcholine receptors. *Grik1* and *Grik4* encode proteins belonging to the kainate family of glutamate receptors. *Cnr1* encodes the cannabinol receptor 1, and phytocannabinoids interact with the CB1 receptor to exert their function, modulating synaptic transmission and ion channel activity [13]. The Western blot analysis highlighted that the treatment with CBN increases the CB1R expression in NSC-34 cells at all concentrations except for the dose of 100 µM. Furthermore, the expression levels of the cannabinoid receptor were more significant at the dose of 5 µM, as reported in Figure 5A. *Gabra5* encodes the alpha 5 subunit of the γ-aminobutyric acid (GABA_A_) receptor. GABA is the major inhibitory neurotransmitter in the CNS; GABA binding to its receptor leads to the influx of Cl^−^ ions, resulting in membrane hyperpolarization and neuronal inhibition [66]. Several studies suggest that GABA and its receptors play a role in the CNS development [67], proliferation, and differentiation of neuronal progenitors, and adult neurogenesis [68,69,70]. *Htr1b* encodes the subunit beta 1 of the 5-hydroxytryptamin receptor, important in memory processing, and whose higher transcriptional levels have been associated with depression, bipolar disorders, and schizophrenia [71]. *Kcnk1* encodes a member of the tandem pore domain potassium channel family. Potassium channels are typically known as “leak channels”, maintain the negative membrane potential, and play a role in excitable cells such as cardiomyocytes, neurons, and muscle cells [72]. Lastly, the *Snc8a* gene encodes the alpha subunit of the voltage-gated sodium channel (Nav1.6) and plays a role in the initiation and propagation of neuronal action potentials. *Snc8a* dysfunction is linked to neurological deficits such as epilepsy and other neurodevelopmental disorders [73,74]. Each gene was differentially expressed at all concentrations of CBN except one. Among all concentrations tested from 5 to 100 µM, the only at which all genes mentioned above resulted in DEGs was the 5 µM dose.

To better understand which dose had a greater impact on synaptic activity, we still filtered the class of DEGs composed by all the genes that showed up as deregulated in at least two comparisons and that, from a certain dose or up to a certain dose, are up- or down-regulated. The analysis highlighted that seven DEGs were differentially expressed in at least two comparisons. In particular, *Cacng8*, *Slc17a7*, and *Tspoap1* were up-regulated, starting from the dose of 20 µM up to 100 µM. *Kcnd2* was up-regulated at the doses of 50 µM and 100 µM. *Cdk5* and *Kcnj4* were up-regulated at the doses 5 µM and 10 µM. At least, *Chrnb4* was down-regulated from the doses of 5 µM to 20 µM.

*Cacng8* encodes the regulatory protein of AMPA receptors, TARP γ-8, also known as calcium voltage-gated channel auxiliary subunit gamma 8. TARP γ-8 plays a critical role for basal expression, trafficking, localization of the AMPA receptor during synaptic development, and plasticity [75]. The *Slc17a7* gene encodes the vesicular glutamate transporter 1 (VGLUT1). VGLUTs are presynaptic components of glutamatergic synapses and regulate synaptic activity through glutamate storage and release into the synaptic cleft [76].The *Tspoap1* gene encodes peripheral-type benzodiazepine receptor-associated protein 1, which exerts its function in a variety of physiological processes, including cholesterol transport and steroid hormone synthesis, mitochondrial permeability and respiration, apoptosis, proliferation, tumorigenesis, and inflammation [77]. The protein expression level of TSPO increases during neuroinflammation or microglia activation, for this reason, TSPO is now widely used as a marker for neuroinflammatory disorders [78]. Furthermore, TSPO ligands have been shown to have anti-inflammatory effects in the CNS and in the peripheral nervous system [78]. Accordingly, TSPO is regarded as an interesting pharmacological target for diagnostic imaging and anti-inflammatory, neuroprotective drug design for the treatment of neurological disorders [79]. *Cdk5* encodes the cyclin-dependent kinase 5 (CDK5), which does not directly regulate the cell cycle; rather, it is widely expressed in postmitotic neurons of the CNS and is mainly involved in neurogenesis, migration and differentiation, axonal and neurite growth, axonal guidance, synaptic plasticity, synaptogenesis, neurotransmission, and apoptosis by phosphorylating key proteins [80]. The *Kcnd2* gene encodes the voltage-dependent potassium channel (Kv4.2), and *Kcnj4* codes for voltage-gated potassium channel subfamily J member 4, known as Kir2.3. The first voltage-gated potassium channel is a major predominant transient outward potassium channel [81], while the second one is classified within the inward rectifier potassium channel family [82]. The *Chrnb4* gene encodes the beta 4 subunit of the cholinergic acetylcholine receptors.

The transcriptomic analysis on NSC-34 cells exposed for 24 h with 5, 10, 20, 50, and 100 µM concentrations of CBN highlighted that all doses used of CBN can strongly alter the processes related to three MFs. Surprisingly, these MFs were related to channel activity, and they were “ion channel activity”, “channel activity”, and “passive transmembrane transporter activity”. Considering all the DEGs without any filter based on the fold change, only one MF was over-represented in every comparison: “ion channel activity”. The DEGs related to “ion channel activity” were 195, and 85 were involved in synaptic activity at different levels. By analyzing the correlated terms and subterms with synaptic activity, we saw that CBN at each dose was able to influence the expression of genes belonging to “synapse”, “glutamatergic synapse”, “postsynaptic membrane”, and “presynaptic membrane”. The transcriptomic analysis shows that each dose is able to influence the gene expression of synaptic activity. Moreover, the fold change values reported for the genes analyzed did not have an increasing or decreasing trend from 5 to 100 µM. In line with the above, we suggest that CBN does not influence the expression of genes involved to synaptic activity in a dose-dependent manner. Since the gene expression levels are comparable in all tested concentrations, we suggest that the concentration of 5 μM is already sufficient to influence the expression of genes involved in synaptic and ion channel activities.

Overall, CBN at concentrations of 5, 10, 20, 50, and 100 µM was able to strongly deregulate gene expressions associated with synaptic and ion channel activity in undifferentiated NSC-34 cells. The affected DEGs had the same expression trend for all concentrations, but the trend was not dose-dependent, and CBN at the concentration of 5 μM was sufficient to induce the regulation of genes related to synaptic and ion channel. CBN was able to regulate the genes coding for different subunits of the calcium, and potassium voltage-gated channels, different subunits of the cholinergic acetylcholine receptors, ionotropic glutamate receptors, γ-aminobutyric acid receptor, 5-hydroxytryptamin receptor, and cannabinol receptor.

Analysis performed using CBN treatment at different doses revealed the alterations in expression of the genes involved in ion activities in a physiological condition. It would be great to carry out additional studies aimed at investigating how the same treatment could act in a clinical condition using an in vitro model. Considering the correlation among the genes involved in the ontologies analyzed and different neurological diseases, is plausible to think that CBN could have some effect in these pathologies. Therefore, additional experiments may be needed, aimed at investigating how the ion influx and neurotransmitter release change with and without the phytocompound. Moreover, the transcriptomic analysis permits an overview of genes mainly influenced by CBN, allowing a more exhaustive evaluation of complex biological processes. Despite being able to identify the genes most perturbed by the phytocompound, the precise mechanism of action of CBN to modify gene expression involved in synapses and ion channel activity is still unclear. Thus, it would be great to explore the changes in gene expression in the presence and absence of different antagonists to understand the molecular mechanism by which CBN acts, and to validate and better characterize our results.

## 5. Conclusions

In this preliminary study, we highlighted that CBN, at each concentration tested, can alter the expression of genes involved in synapses and ion channel activity. Expression of the genes discussed assumes the same trend, up- or down-regulated, regardless of the dose of CBN used for the treatment. In particular, considering the voltage-gated calcium channel subunits, as a result of the treatment with CBN, the expression of *Cacna1b*, *Cacna1h*, and *Cacng8* increased, while the expression of *Cacnab4* and *Cacng2* decreased. Regarding potassium influx, the expression of the genes coding for voltage-gated potassium channel subunits (*Kcnc3*, *Kcnd1*, *Kcnd2*, and *Kcnj4*) increased after the CBN treatment. CBN treatment resulted also in the up-regulation of the genes involved in the glutamatergic and GABAergic pathways (*Grik5*, *Grik1*, *Slc17a7*, *Gabra5*) and in the down-regulation of genes involved in the serotoninergic and cholinergic pathways (*Htr3a*, *Htr3b*, *Htr1b*, *Chrna3*, *Chrnb2*, *Chrnb4*). Based on our transcriptomic results, CBN could be considered a potential compound that influences the ion influx and consequentially neurotransmitter release in excitable cells. In this regard, further studies are needed to observe its effect in a neurological disease model. The genes discussed show a comparable fold change at each dose of CBN, so we can confirm that the lowest dose used, CBN 5 μM, is enough to generate an imbalance at transcriptomic levels comparable to the higher doses of CBN used.

## Figures and Tables

**Table 1:** Number of DEGs with an extreme fold change and over-represented ontologies.

Comparisons	No. DEGs			No. BPs	No. MFs	No. CCs
	fc ≤ −2	fc ≥ 2	Tot			
CTRL vs. CBN 5 µM	348	580	928	0	23	9
CTRL vs. CBN 10 µM	345	593	938	84	11	4
CTRL vs. CBN 20 µM	425	442	867	0	7	0
CTRL vs. CBN 50 µM	365	607	972	40	7	12
CTRL vs. CBN 100 µM	416	472	888	16	6	13

In the first column are reported the different comparisons performed. Following the comparisons are reported the number of DEGs with fold change ≤−2 or ≥2 and the sum of these elements. In the last three columns are reported the number of the gene ontologies over-represented for the different comparisons including just the DEGs with extreme fold change.

**Table 2:** Fold change of the genes resulting in DEGs in all comparison.

DEGs	CTRL vs. CBN 5 µM	CTRL vs. CBN 10 µM	CTRL vs. CBN 20 µM	CTRL vs. CBN 50 µM	CTRL vs. CBN 100 µM
*Cacna1b*	0.25	0.16	0.21	0.10	0.30
*Cacna1h*	0.40	0.47	0.31	0.33	0.42
*Cacnb4*	−2.10	−1.18	−1.30	−4.29	−2.85
*Cacng2*	−0.33	−0.54	−0.81	−0.71	−0.72
*Gjc1*	−0.28	−0.21	−0.27	−0.52	−0.58
*Grik5*	0.21	0.26	0.10	0.10	0.08
*Grin1*	−0.17	−0.35	−0.39	−0.28	−0.14
*Htr3a*	−0.32	−0.85	−0.63	−0.64	−0.70
*Htr3b*	−5.64	−1.86	−5.16	−3.38	−5.39
*Kcnc3*	0.65	0.58	0.66	0.65	0.60
*Kcnd1*	1.01	1.66	1.36	0.94	1.38
*Snap25*	−0.13	−0.10	−0.12	−0.10	−0.17

The first column reports the gene list and in the other columns are reported the fold changes of all the genes involved in the terms and sub terms highlighted in Figure 4 and resulting in DEGs in all the comparison with the same trend across them.

**Table 3:** Fold change of the genes resulting in DEGs in four out of five comparisons.

DEGs	CTRL vs. CBN 5 µM	CTRL vs. CBN 10 µM	CTRL vs CBN 20 µM	CTRL vs. CBN 50 µM	CTRL vs. CBN 100 µM
*Chrna3*	−0.38	-	−0.25	−0.19	−0.23
*Chrna4*	5.95	4.32	-	5.23	5.30
*Chrnb2*	−0.13	−0.07	−0.27	−0.22	-
*Cnr1*	0.11	0.17	0.27	-	0.12
*Gabra5*	6.16	-	6.05	5.06	5.08
*Grik1*	0.81	1.33	1.00	0.91	-
*Grik4*	−1.23	-	−0.47	−1.04	−0.52
*Htr1b*	−4.19	−2.44	-	−4.38	−2.95
*Kcnk1*	−1.56	−1.59	−3.67	-	−0.99
*Scn8a*	0.13	0.24	-	0.27	0.22

The first column reports the gene list, and in the other columns are reported the fold change of all the genes involved in the terms and subterms highlighted in Figure 4, which were DEGs in four out of five comparisons. Cells filled with “-” indicates that for that comparison, the gene does not results in DEGs.

**Table 4:** Fold change of the genes with an ascending or descending trend across the comparison.

DEGs	CTRL vs. CBN 5 µM	CTRL vs. CBN 10 µM	CTRL vs. CBN 20 µM	CTRL vs. CBN 50 µM	CTRL vs. CBN 100 µM
*Cacng8*	-	-	0.36	0.37	0.48
*Kcnd2*	-	-	-	0.31	0.45
*Slc17a7*	-	-	0.87	0.53	0.77
*Tspoap1*	-	-	1.48	1.2	1.05
*Cdk5*	0.17	0.19	-	-	-
*Chrnb4*	−0.87	−0.33	−0.94	-	-
*Kcnj4*	2.89	2.36	-	-	-

The first column reports the gene list, and in the other columns are reported the fold change of all the genes involved in the terms and subterms highlighted in Figure 4 and resulting in DEGs in at least two out of five comparisons with the same trend across them. The reported DEGs show an increment or decrement in the expression until to or starting from a certain dose. Cells filled with “-” indicate that for that comparison, the gene does not result in DEGs.

## Data Availability

The data presented in this study are openly available in the NCBI Sequence Read Archive at BioProject accession number PRJNA1144250.

## Supplementary Materials

The following supporting information can be downloaded at: https://www.mdpi.com/article/10.3390/cells13181573/s1, Table S1: all transcripts, Table S2: ORA including extreme fold change DEGs, Table S3: ORA including all the DEGs, Table S4: DEGs involved in the MF “ion channel activity”, Table S5: DEGs involved in synaptic activity, Table S6: DEGs involved in “synapse”, “glutamatergic synapse”, “postsynaptic membrane”, and “presynaptic membrane”, Figure S1: Western blot results for CB1R, and Figure S2: Western blot results for NMDAR2B.
