# Supplementary material for: Cannabinol (CBN) Influences the Ion Channels and Synaptic-Related Genes in NSC-34 Cell Line: A Transcriptomic Study
Source: Cells. 2024 Sep 19;13(18):1573. doi: 10.3390/cells13181573 (PMC11430194; doi:10.3390/cells13181573)
Supplement: Supplementary file 1 [file cells-13-01573-s001.zip › cells-3176121-supplementary-Figures.pdf]

Western Blot results for CB1R

In the following section we report the Western blot gels for CB1R.

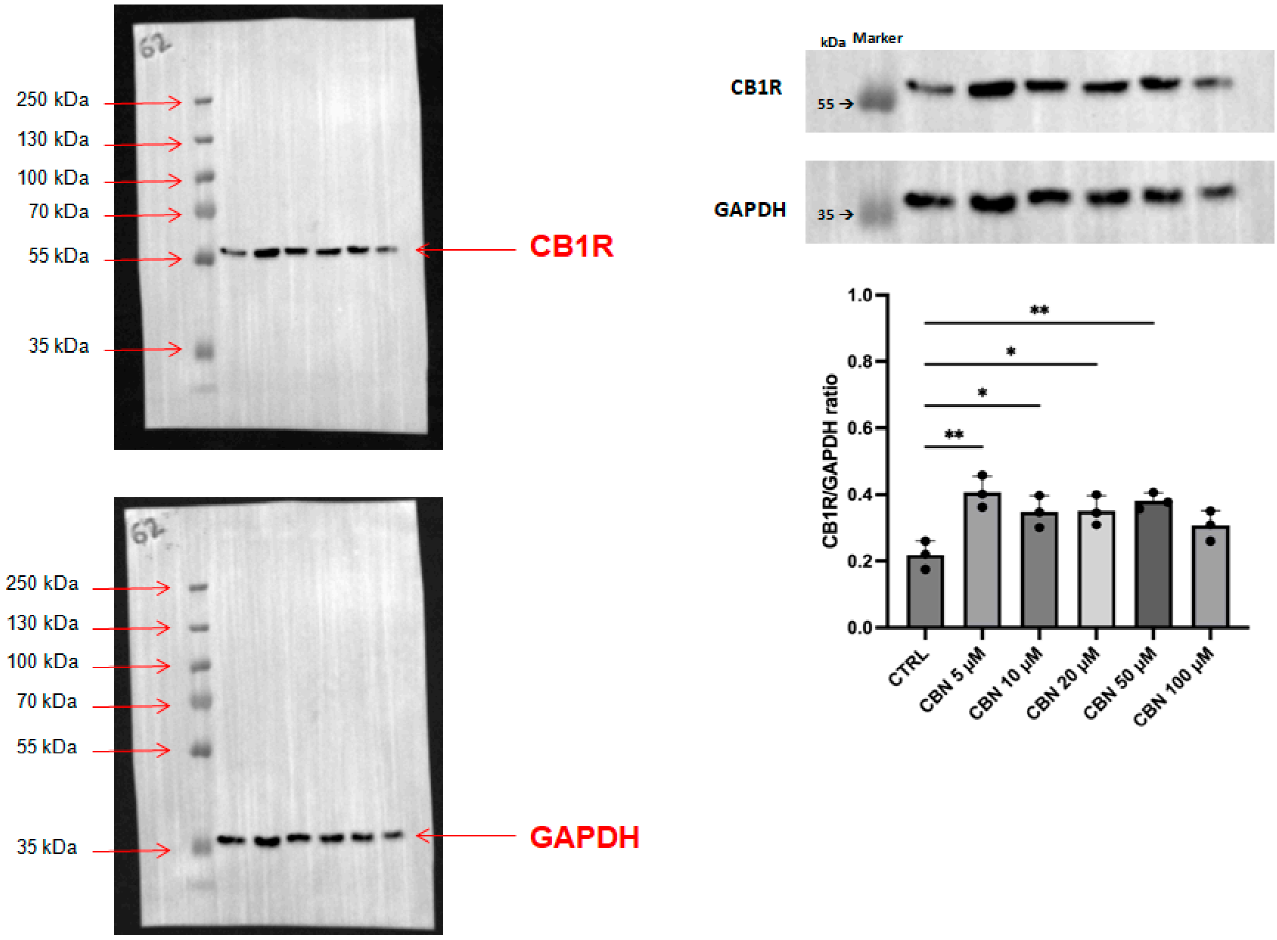

Figure S1. Here, we report the results obtained from western blot analysis of CB1R expression evaluated on the whole protein fraction. On the left-hand side of the figure, we report the original pictures of the membranes of CB1R and GAPDH. GAPDH was used as housekeeping protein to normalize the absorbance values of CB1R at the various tested CBN conditions. For CB1R a significant increase of protein expression compared to control was highlighted at CBN concentrations from 5 µM to 50 µM. This result suggests the CBN ability to induce an up-regulation on CB1R protein expression.

Western Blot results for NMDAR2B

In the following section we report the Western blot gels for NMDAR2B.

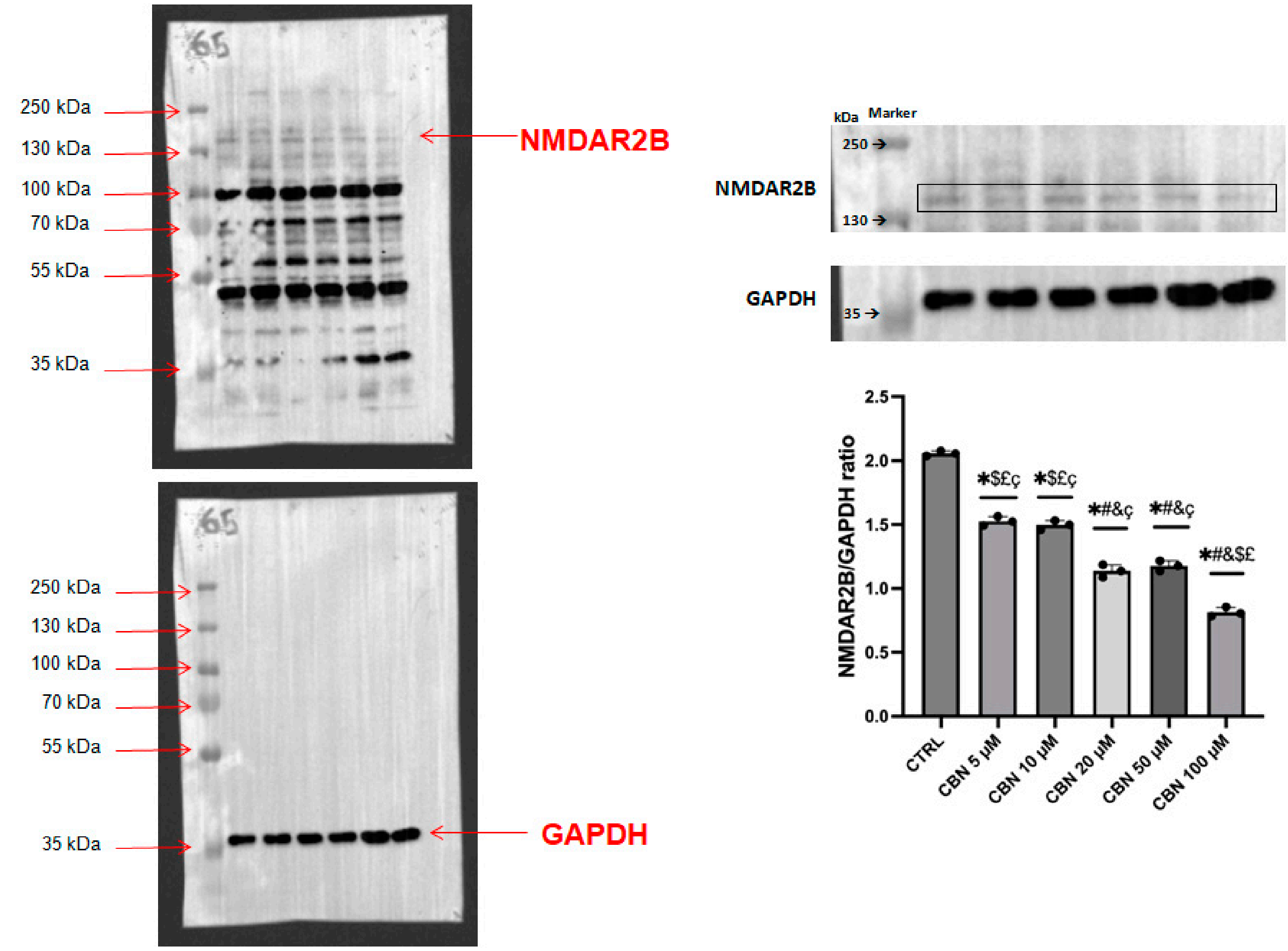

Figure S2. Here, we report the results obtained from western blot analysis of NMDAR2B expression evaluated on the whole protein fraction. On the left-hand side of the figure, we report the original pictures of the membranes of NMDAR2B and GAPDH. GAPDH was used as housekeeping protein to normalize the absorbance values of NMDAR2B at the various tested CBN conditions. For NMDAR2B a significant decrease of protein expression compared to control was highlighted at all tested CBN concentration. Interestingly, the CBN treatment effect in down-regulating NMDAR2B protein expression increased progressively with increasing dose compared to control. Overall, these results suggest that CBN is capable to induce a down-regulation on NMDAR2B protein expression.
