# Supplementary material for: Cannabinol (CBN) Influences the Ion Channels and Synaptic-Related Genes in NSC-34 Cell Line: A Transcriptomic Study
Source: Cells. 2024 Sep 19;13(18):1573. doi: 10.3390/cells13181573 (PMC11430194; doi:10.3390/cells13181573)
Supplement: Supplementary file 1 [file Cover letter.pdf › Cover letter.pdf]

CENTRO
NEUROLESI
**BONINO
PULEJO**
IRCCS MESSINA

Istituto di Ricovero e Cura
a Carattere Scientifico

UNI EN ISO 9001:2008 CERTIQUALITY SISTEMA DI GESTIONE QUALITÀ CERTIFICATO
CERTIQUALITY È MEMBRO DELLA FEDERAZIONE CISQ IQNet
Certificato n. 10271

Italy, 19th June 2023

Dear Editor,

we would like to propose the enclosed manuscript entitled "Transcriptome highlights cannabinol modulation of mitophagy in a Parkinson's disease in vitro model" as possible publication in Biomolecules in the special issue "Mitochondrial Quality Control in Aging and Neurodegeneration".

Parkinson's disease (PD) is a neurodegenerative disorder characterized by the loss of dopaminergic neurons in the substantia nigra and the accumulation of Lewy bodies. Mitochondria dysfunctions, included an impaired mitophagy, are common features of PD. Cannabinoids are arising as new therapeutic strategies against neurodegenerative diseases. In this study, we evaluated the potential protective effects of cannabinol (CBN) pre-treatment in an in vitro PD model through a transcriptomic analysis using Next Generation Sequencing (NGS). CBN was able to counteract the loss of cell viability induced by MPP^+ treatment. Focusing on biological processes relative to mitochondria functions, we found that CBN pre-treatment can attenuate the expression of genes involved in biological processes relative to mitochondria transport, localization and protein targeting. Moreover, MPP^+ treatment increased the expression of the genes involved in PINK1/Parkin mitophagy, while CBN reduced their expression.

The manuscript has never been published, and is not under consideration for publication elsewhere. No conflict of interest has to be declared. All other Authors have read the manuscript and have agreed to submit it in its current form for consideration for publication in the Journal. We strongly hope that this could be a good job, in accordance with the expectations of your journal and worthy to be published.

Sincerely yours,

Emanuela Mazzon
IRCCS - Centro Neurolesi
"Bonino-Pulejo", Messina (Italy)
